# The projected economic burden and complications of revision hip and knee arthroplasties: Insights from national registry studies

**DOI:** 10.1002/ksa.12678

**Published:** 2025-04-13

**Authors:** Patrick Sadoghi, Amir Koutp, Daniel Perez Prieto, Martin Clauss, M. Enes Kayaalp, Michael T. Hirschmann

**Affiliations:** ^1^ Department of Orthopaedics and Trauma Medical University of Graz Graz Austria; ^2^ Orthopedic Department, Septic Unit, Hospital del Mar Universitat Pompeu Fabra Barcelona Spain; ^3^ IcatKnee. Hospital Dexeus Barcelona; ^4^ Department for Orthopaedics and Trauma Surgery University Hospital Basel Basel Switzerland; ^5^ Center for Muskuloskeletal Infections (ZMSI) University Hospital Basel Basel Switzerland; ^6^ Department of Orthopaedics and Traumatology, University of Health Sciences Istanbul Fatih Sultan Mehmet Training and Research Hospital Istanbul Turkey; ^7^ Department of Orthopedic Surgery and Traumatology Kantonsspital Baselland, Bruderholz Binningen Switzerland; ^8^ Department of Clinical Research, Research Group Michael T. Hirschmann, Regenerative Medicine & Biomechanics University of Basel Basel Switzerland

**Keywords:** aseptic loosening, hip arthroplasty, knee arthroplasty, national registry, septic complications

## Abstract

The rising volume of primary hip and knee arthroplasties has led to a parallel increase in revision surgeries, creating significant clinical and economic challenges for healthcare systems worldwide. This study synthesizes national arthroplasty registry data to evaluate trends in revision aetiology, associated costs and regional disparities. While advancements in prosthetic design have reduced aseptic loosening rates (declining to 35.1% for hips and 18.3% for knees), septic complications now account for a growing proportion of revision cases, rising to 18.2% for hips and 21.6% for knees. Additionally, instability and malalignment persist at 15.9% and 14.1%, respectively. Revision procedures are 76% more costly than primary surgeries, with two‐stage septic revisions incurring costs of up to $37,297 per case. Beyond direct surgical costs, prolonged recovery and productivity loss contribute to a broader economic impact. Regional variations, such as higher periprosthetic fracture rates in England and Wales, highlight inconsistencies in data reporting and healthcare practices. Addressing these challenges requires standardized infection definitions, enhanced registry collaboration and investment in infection prevention strategies. The role of referral centres in improving outcomes and reducing costs through multidisciplinary care is increasingly recognized. By integrating evidence‐based infection management protocols and leveraging emerging technologies, the orthopaedic community can optimize patient outcomes and reduce the financial burden of revising arthroplasties.

**Level of Evidence:**

Level IV.

**Abbreviations**
EBJIS: European Bone and Joint Infection Society
MSIS: Musculoskeletal Infection Society
PJI: periprosthetic joint infection
THA: total hip arthroplasty
TKA: total knee arthroplasty

## DATA SOURCES AND LIMITATIONS

This analysis is based on data extracted from national arthroplasty registries, which systematically document revision rates, causes of failure, and economic impact. These registries provide robust datasets for evaluating long‐term trends; however, variations in reporting criteria pose inherent limitations. A key challenge lies in the inconsistent definitions of PJI across registries. While some datasets adhere to the Musculoskeletal Infection Society (MSIS) criteria, others follow the European Bone and Joint Infection Society (EBJIS) guidelines, leading to discrepancies in reported infection rates. Additionally, economic assessments differ in how direct and indirect costs are calculated, further complicating cross‐registry comparisons. Despite these limitations, the findings presented here synthesize data from sources with rigorous classification frameworks, providing a comprehensive overview of revision arthroplasty trends. Moving forward, efforts to standardize registry definitions and harmonize reporting methods will be crucial for enhancing data accuracy and comparability.

### The growing demand for hip and knee arthroplasties: Trends and complexities

The demand for total hip arthroplasty (THA) and total knee arthroplasty (TKA) has increased exponentially due to the ageing global population, rising rates of osteoarthritis, and expanded access to healthcare services. Trends indicate a significant rise in arthroplasty procedures worldwide, driven by the increasing prevalence of osteoarthritis and demographic changes [23, 52]. Recent analyses of global registries have provided valuable insights into advancements in surgical techniques for TKA and the improving survival rates of THA [3, 15, 16, 25].

For instance, the number of TKA procedures in the United States is projected to increase by 182% by 2030, with annual total knee replacements expected to reach 3.5 million [45]. While precise global data are lacking, these localized trends reflect a broader worldwide demand for joint replacement surgery. Surgical innovations, including improved prosthetic materials, enhanced osseointegration techniques and minimally invasive procedures, have led to better outcomes but have also introduced new complexities.

Revision surgery presents a significant challenge due to its technical demands and the associated financial and clinical burdens [47]. While aseptic loosening was historically the leading cause of revisions, registry data now indicate a shift toward septic complications and instability. Addressing these challenges requires both innovative solutions and a reassessment of current practices and postoperative practices [17, 23].

Additionally, demographic factors are playing an increasingly critical role. Candidates for joint replacement are often older and more likely to have comorbidities such as diabetes, obesity or cardiovascular disease, all of which elevate the risk of complications [33, 46, 53]. This underscores the need for customized prevention strategies and comprehensive perioperative care to optimize patient outcomes.

### Septic complications in hip versus knee arthroplasties: A rising concern

One of the most concerning trends over the past decade is the increasing prevalence of septic complications as the leading cause of TKA revision procedures. National registry data indicate that infection‐related revisions have risen from 14.8% to 21.6% for knee replacements and from 7.5% to 18.2% for hip replacements [17, 41]. Despite the widespread use of perioperative antibiotics and strict infection control measures, these numbers continue to rise, although it may be a consequence of a better diagnostic algorithm and also better data collection of the registries [27, 32, 48]. A recent analysis highlights the significant risks associated with failed hip and knee replacements due to PJIs and underscores the long‐term challenges these complications present [24]. Similarly, a study by Schroer et al. identified infection as one of the primary causes of failure in modern knee arthroplasty, emphasizing the substantial burden of septic complications [44].

Several factors contribute to this trend, including an ageing population with higher comorbidity rates, the increasing use of immunosuppressive therapies and the emergence of multidrug‐resistant organisms [33]. Additionally, as previously mentioned, studies suggest that PJI is underreported due to inconsistent documentation in various registries [19, 58]. Standardized definitions, optimal diagnostic tools and improved data collection practices are urgently needed to accurately assess and address the true extent of this problem.

Emerging technologies, such as advanced microbial detection systems, real‐time imaging and machine learning algorithms, hold promise for revolutionizing early diagnosis and risk stratification. These innovations could significantly enhance infection prevention, diagnostics, management and treatment outcomes [13, 14, 43, 55].

### The decline of aseptic loosening in hip versus knee revisions: Advances and opportunities

While septic complications are on the rise, aseptic loosening has seen a significant decline, largely due to innovations in prosthetic design and surgical techniques. The number of revisions attributed to aseptic loosening decreased from 55.2% to 35.1% for THA and from 29.8% to 18.3% for TKA [17, 25, 41]. Key contributing factors include improved osseointegration, reduced wear rates and enhanced fixation methods [26, 36].

Additionally, the decline may reflect advancements in diagnostic tools that more accurately differentiate aseptic loosening from other causes, such as infection or malalignment [51]. More investments in materials science—particularly in bioactive coatings and anti‐inflammatory surfaces—will be essential to sustaining these positive results in the future.

### Instability and malalignment: Comparing hip and knee revision challenges

Instability and malalignment remain significant concerns in joint arthroplasty revisions, with registry data indicating an increase in revisions due to instability from 11.8% to 15.9% in THA and from 6.2% to 14.1% in TKA [17, 41]. Contributing factors include improper implant positioning, soft tissue imbalance and inadequate rehabilitation [56].

The integration of advanced surgical planning tools, such as robotic‐assisted systems and patient‐specific instrumentation, has been shown to improve implant positioning accuracy, which is directly associated with lower revision rates in registry studies [11, 57]. A recent analysis of registry data suggests that malalignment‐related revisions are significantly lower in procedures utilizing robotic‐assisted systems due to enhanced precision in component placement and soft tissue balancing [49].

Additionally, 3D printing technology enables the development of customized implants and surgical guides, which is particularly beneficial in complex revision cases where standard implants may not provide an optimal fit. Registry‐based studies indicate that patient‐specific 3D‐printed implants improve osseointegration and reduce revision rates associated with component misalignment [11, 57].

Furthermore, reducing surgical time through patient‐specific instrumentation has been correlated with lower intraoperative complications and improved early functional outcomes [49].

By leveraging these technological advancements, surgeons can minimize malalignment‐related failures, improve long‐term implant survival and reduce the burden of revision surgeries on healthcare systems.

### Regional variations in hip and knee revision cause: Data inconsistencies

National registries reveal significant regional differences in the causes of revision surgeries. For example, lower infection rates but higher rates of periprosthetic fractures have been reported in England, Wales and Northern Ireland compared to other regions [2, 4, 12, 28, 29, 42, 50]. These discrepancies may be attributed to multiple factors, including differences in surgical techniques, implant selection, patient demographics (e.g., osteoporosis prevalence or obesity rates) and registry data reporting standards.

While standardized definitions for infection, such as those established by the MSIS and the EBJIS, are already in place, there remains an urgent need for standardized outcome measures to improve global comparability and enhance clinical research. Moreover, registry differences in capturing periprosthetic fracture incidence—ranging from radiographic confirmation to symptomatic fractures requiring revision—further complicate cross‐country comparisons.

Addressing these variations requires enhanced international collaboration, adoption of harmonized reporting standards and integration of big data analytics to refine risk factor identification and optimize best practices in revision arthroplasty management [27, 32, 37].

### Economic burden of hip versus knee revision arthroplasties: A cost analysis

Revision arthroplasties pose not only a clinical challenge but also a significant economic burden. Revision surgery is, on average, 76% more expensive than primary joint replacement, with the direct costs for revision hip surgery reaching $14,813 and up to $37,297 for two‐stage replacement procedures [5, 54]. Medicare reimburses physicians an average of $1499.89 per knee revision and $1603.32 per hip revision, highlighting the immense financial impact on the healthcare system [18]. Similarly, in Europe, the total healthcare reimbursement for PJIs following primary hip and knee arthroplasty in 2019 was estimated to be €346,262,026, with hip PJIs accounting for €197,230,953 and knee PJIs for €149,031,073 [1]. Beyond direct medical expenses, revision surgeries impose substantial indirect costs, including lost productivity, prolonged recovery periods, caregiver burden and diminished quality of life. A recent study found that indirect costs can surpass direct surgical expenses in patients with complex PJIs, especially in working‐age populations [30]. Studies suggest that treating infections in high‐volume referral centres may lower costs due to standardized protocols and a multidisciplinary treatment approach, particularly in multi‐revised infection cases [7, 10]. Ferguson et al. demonstrated that centralized infection management in specialized centres reduces complication rates, shortens hospital stays and improves long‐term functional outcomes, reinforcing the economic benefits of these institutions [6, 8, 9].

However, cost savings vary regionally depending on healthcare infrastructure, reimbursement policies and access to specialized centres. For example, data from centralized healthcare systems in Scandinavia suggest that structured revision programmes lead to improved cost efficiency over time, while fragmented healthcare models in other regions may experience higher long‐term costs due to inconsistent referral pathways and prolonged hospital stays.

To mitigate these economic challenges, policymakers must prioritize funding for infection prevention programmes, optimize referral centre networks and invest in surgical training initiatives. Early intervention strategies—including enhanced preoperative screening, patient education and optimized perioperative care—can significantly reduce both direct and indirect costs, alleviating the burden on healthcare systems.

### Recommendations and future directions

To address the growing burden of revision arthroplasties, the orthopaedic community must implement evidence‐based strategies:

1. **Standardize PJI Definitions:** Implement criteria from the MSIS and EBJIS to improve data consistency and accuracy across registries.
2. **Enhance Data Collection Practices:** Develop comprehensive reporting systems, train data entry personnel and promote registry standardization to improve accuracy and comparability.
3. **Refine Surgical Techniques:** Expand surgeon education programmes, adopt robotic‐assisted surgery and integrate preoperative planning tools to reduce complication rates and optimize implant placement.
4. **Conduct Region‐Specific Research:** Investigate geographic disparities in revision arthroplasty outcomes by analyzing variations in surgical techniques, patient demographics, and healthcare policies to inform best practices.
5. **Leverage Predictive Analytics and Technological Innovations**: Utilize machine learning algorithms to analyze registry data and identify early risk factors for revision surgery, including patient demographics, implant characteristics and surgical variables. Implement AI‐based infection risk prediction models to stratify patients preoperatively and guide prophylactic measures. Incorporate 3D printing and bioactive materials to improve implant integration and longevity in high‐risk revision cases.
6. **Strengthen Infection Prevention Protocols:** Adopt multidisciplinary approaches and expand antimicrobial stewardship programmes.
7. **Establish Unified Outcome Measures:** To enhance global comparability in revision arthroplasty research, defining standardized outcome measures is essential. Key metrics should include (1) patient‐reported outcome measures (PROMs), such as the Oxford Knee Score (OKS) and Hip Disability and Osteoarthritis Outcome Score (HOOS); (2) functional outcomes, including range of motion and implant survival at 1, 3 and 5 years post‐revision; (3) infection eradication rates following PJI treatment; and (4) healthcare utilization metrics, such as hospital length of stay, readmission rates and re‐revision incidence. Implementing these measures across national registries would allow for better cross‐country benchmarking and quality assessment in revision arthroplasty research.

## INTRODUCTION

Arthroplasty registries serve as systematic databases for documenting and monitoring joint replacement surgeries, offering invaluable insights into implant performance, patient outcomes, and revision trends [35]. These registries facilitate comparisons of implant survival rates, identify at‐risk populations and provide essential data for improving surgical decision‐making and healthcare policies [20, 21, 23, 25, 37, 38]. With over 1.5 million total hip and knee replacements performed annually in the United States alone, the importance of registry data has never been greater [31]. While advancements in prosthetic materials and surgical techniques have significantly improved implant longevity and patient outcomes, the rising global demand for joint replacements has also led to a corresponding increase in revision surgeries [3, 23, 25, 34, 40]. These revisions are driven by complications such as aseptic loosening, instability and periprosthetic joint infections (PJIs) [17, 26, 36, 39]. Among these, septic complications have become an increasingly prevalent cause of failure, often requiring complex and costly interventions, including multiple surgeries and long‐term antimicrobial therapy [17, 22]. Despite the wealth of data available from national registries, inconsistencies in reporting and definitions—such as variations in PJI classification criteria—pose challenges for cross‐registry comparisons. Furthermore, economic analyses of revision procedures remain underdeveloped, with limited standardization in assessing direct and indirect costs.

This editorial aims to synthesize insights from national arthroplasty registries to examine key trends in revision aetiology, assess the economic burden of revision surgeries, and propose evidence‐based strategies for prevention, resource optimization and future research. By addressing both clinical and economic challenges, this analysis seeks to inform strategies that can mitigate the rising burden of revision arthroplasties globally.

## AUTHOR CONTRIBUTIONS

**Patrick Sadoghi**: Supervision; conceptualization; data curation; writing—review and editing. **Amir Koutp**: Investigation; methodology; writing—review and editing. **Daniel Perez Prieto**: Conceptualization; data curation; writing—review and editing. **Martin Clauss**: Investigation; data curation; writing—review and editing. **M. Enes Kayaalp**: Conceptualization; data curation; writing—review and editing. **Michael T. Hirschmann**: Conceptualization; data curation; writing—review and editing.

## CONFLICT OF INTEREST STATEMENT

Michael T. Hirschmann: Consulting fees from Depuy Synthes and Symbios; Honoraria for Lectures and Support attending Meetings from Depuy Synthes, Symbios, and S&N; Participation on Advisory Board for Depuy Synthes; Leadership positions in *KSSTA Journal*, *ESSKA*, *German Knee Society* and *Personalized Arthroplasty Society*. Patrick Sadoghi: Industry grants from DePuy Synthes, Johnson & Johnson, alphamed and Medacta; Editorial Board Member for *JOA*, *KSSTA* and *Arthroscopy*. M. Enes Kayaalp: ESSKA U‐45 Committee; KSSTA Associate Editor; JOA Editorial Board Member. The remaining authors declare no conflicts of interest.

## ETHICS STATEMENT

This study complies with ethical standards. Informed consent was obtained where applicable.

## CONCLUSION

The increasing prevalence of septic complications and instability in revision arthroplasty signals a critical shift in joint replacement surgery. While advancements in prosthetic technology have reduced aseptic loosening, infection management and implant durability remain major challenges. Addressing these issues requires standardized infection definitions, improved registry harmonization and investment in high‐volume referral centres.

Beyond clinical challenges, the financial burden of revision arthroplasty necessitates urgent policy action. Healthcare decision‐makers must prioritize funding for infection prevention, expand surgical training initiatives and promote cost‐effective care pathways, particularly through centralized infection management strategies proven to reduce complications.

By integrating predictive analytics, AI‐driven infection risk assessment and 3D‐printed implants, alongside robust infection control measures, the orthopaedic community can mitigate the clinical and economic burden of revision surgeries and improve long‐term patient outcomes.

## Data Availability

Data supporting the findings of this study are available upon reasonable request from the corresponding author.
